# First description of the larva of *Dinaraea* Thomson, 1858, with comments on chaetotaxy, pupa, and life history based on two saproxylic species from Europe (Staphylinidae, Aleocharinae, Athetini)

**DOI:** 10.3897/zookeys.752.24440

**Published:** 2018-04-23

**Authors:** Bernard Staniec, Ewa Pietrykowska-Tudruj, Krzysztof Pawlęga

**Affiliations:** 1 Department of Zoology, Maria Curie-Sklodowska University, Akademicka 19, 20-033 Lublin, Poland; 2 Department of Zoology, Ecology and Wildlife Management, University of Life Sciences in Lublin, Akademicka 13, 20-950 Lublin, Poland

**Keywords:** *
aequata*, aleocharines, Coleoptera, developmental stages, early and late larval instars, ecological preferences, *
linearis*, morphology, pupal stage, rove beetles, staphylinids, subcortical, ultrastructure

## Abstract

The paper describes the morphological ultrastructure of the previously unknown early (L1) and late larval instars (L2–3) of *Dinaraea*, including chaetotaxy, pupal cocoon, prepupa, and pupa, based on the saproxylic species *D. aequata* Erichson and *D. linearis* Gravenhorst. Diagnostic larval characters for the genus *Dinaraea* are given for the first time. Morphological differences between mature larvae of these two species relate to the colouration and degree of flattening of the body, details of antennal structure, anterior margin of the labrum, mandibles, and mala. The differences are relatively small, probably because of the similar ecological preferences of both species. As in the case of other aleocharine larvae, L1 in *Dinaraea* differs from L2–3 in the lack of some setae on the dorsal surface of the head and thorax, and on the abdominal tergites and sternites; the presence of a subapical seta on the urogomphi; egg bursters on some thoracic and abdominal tergites; a darker antennal segment III; and the relatively longer urogomphi and their apical setae. The differences established in the features of the chaetotaxy of L1 and L2–3 between Athetini (*Dinaraea*), Oxypodini (*Thiasophila*) and Homalotini (*Gyrophaena*) correspond with the molecular marker-based relationships of these taxa.

## Introduction

The genus *Dinaraea* Thomson, 1858 (Staphylinidae, Aleocharinae, Athetini) includes 21 species worldwide, 12 of which are known from the Nearctic and nine from the Palaearctic; five of the latter (*D. aequata* Erichson, *D. angustula* Gyllenhal, *D. arcana* Erichson, *D. hungarica* Ádám, *D. linearis* Gravenhorst) occur in Europe. They are small insects (the lengths of the European species are 2.5–3.7 mm) with a subparallel, flattened body, and the integument has a distinct meshed microsculpture and distinct punctation. The head is large, subquadrate to slightly elongate, and the genae are usually longer than the eyes. Mainly saproxylic, *Dinaraea* species inhabit the subcortical galleries of other insects. They are also found in rotting tree trunks and in the fruiting bodies of various polypores. Because of their environmental preferences and their probably predatory mode of life, most species of this genus are potentially important as enemies of economically significant forest pests. To date, however, the diet of these rove beetles remains unknown, as do other aspects of their biology (Benick and Lohse 1974, Nikitsky and Schigel 2004, Klimaszewski et al. 2013, Löbl and Löbl 2015).

Nothing is known of the morphology of the preimaginal stages of *Dinaraea*. This is not particularly surprising, since very little information is available on the external structure of other Athetini taxa, just as is the case with most Aleocharinae. The larvae of only a few of the more than 170 genera classified among Athetini are known (Paulian 1941, Pototskaya 1967, Topp 1978, Ashe and Watrous 1984, Ashe 1985, Newton et al. 2000, Ashe 2005). What is more, such descriptions as do exist are usually fragmentary and relate to just a few features illustrated in diagrams. The very poor state of knowledge regarding the larvae of these staphylinids makes it almost impossible to make use of their morphologies in phylogenetic analyses. Only Ashe (2005), in a work on the phylogeny of the tachyporine group subfamilies and ‘basal’ lineages of the Aleocharinae, took into account features of both imagines and larvae, including three genera of Athetini. It turns out that the larval characters to a large extent stabilise the phylogenetic tree covering the taxa under consideration in the present work. In the case of other aleocharines, this same author also used larval morphologies to examine phylogenetic relationships within the subtribe Gyrophaenina (Ashe 1986). The results turned out to be confluent with the morphological analysis of the imagines of these Staphylinidae. They point to the distinct monophyletic origin of that subtribe and are strongly underpinned especially by the external features of these Aleocharinae. The greater usefulness of larval than imaginal stages in phylogenetic analyses was also demonstrated by Grebennikov and Newton (2009) in the case of ten subfamilies in the Staphylininae group. Again, Pietrykowska-Tudruj et al. (2011, 2014) highlighted the great importance of larval features in establishing the systematic membership of *Quedius antipodum* Sharp and the genus *Astrapaeus* Gravenhorst. The results substantiate data obtained from analyses of adult morphology and/or DNA sequences, and suggest the separate position of *Q. antipodum* in relation to the north temperate genus *Quedius* and the genus *Astrapaeus* within the tribe Quediini.

The necessity to take larval morphological features into consideration in future phylogenetic analyses and assessments of the systematic membership of Staphylinidae thus seems wholly logical. Unfortunately, a major obstacle to doing so is the insufficient and often extremely fragmentary nature of the relevant data, as mentioned above, which applies in particular to the subfamily Aleocharinae, including the tribe Athetini.

The main aim of this study is to describe in detail the external morphology, including the chaetotaxy and ultrastructure, of the early (L1) and late (L2–3) larval instars of *Dinaraea* based on *D. aequata* and *D. linearis*. The paper also includes data on the external appearance of the hitherto unknown pupa of this genus, as well as the feeding preferences and the life histories of both species.

## Materials and methods

Larval and pupal stages of the two species were obtained by rearing five adults of *D. aequata* and four adults of *D. linearis*. Specimens of *D. aequata* were collected at Parchatka near Kazimierz Dolny (51°22'54.55"N, 21°59'51.53"E, SE Poland) on 11 November 2004. The insects were sifted from the remains of birch bark, in deciduous woodland growing in a shady, damp loess gully. Individuals of *D. linearis* were collected at Łańcuchów near Lublin (51°16'15.33"N, 22°55'20.35"E, SE Poland) on 3^rd^ December 2004. These beetles were sifted from pieces of bark torn off a wind-thrown ash (*Fraxinus excelsior* L.) in an old riparian wood of ash and alder (Circaeo-Alnetum) in the valley of the River Wieprz, a dozen or so metres from the river bank. *D. aequata* and *D. linearis* were reared from 19 November 2004 to 20 January 2005 and from 6 December 2004 to 21 February 2005, respectively, at room temperature (20 °C ± 3). Adults and larvae of both species were kept separately in plastic containers (diameter 10 cm, height 2 cm) filled with moist soil. Larvae were fed various sizes of small springtails of the family *Onychiuridae*. The immature stages (larvae and pupae) were killed in boiling water and preserved in ethanol (75%). The adults were identified by the first author.

Morphometry and morphology: specimens were measured using an Olympus BX63 compound microscope. Measurements were made in cellSens Dimension v1.9 software and are given in millimetres. Photographs showing total aspects of the mature (L3) larvae of both species, as well as the prepupa and pupal cocoon of *D. linearis* were taken with an Olympus DP72 digital camera mounted on an Olympus SZX16 compound microscope (Figs 1–6, 61–64). To prepare microscope slides for the morphological analyses, the preserved larvae were treated with 10% KOH for approximately twelve hours, rinsed in distilled water, then immersed in lactic acid. Photographs showing various details of the external structure of larva and the total aspect of larva and pupa were taken using an Olympus DP21 digital camera mounted on an Olympus BX63 compound microscope (Figs 14, 15c, 18–21, 24–26, 28–31, 33–35, 41, 43–60) or with a VEGA3 TESCAN SEM (Figs 7–11, 12, 13a, b, 15a, b, 16, 22, 23, 27a, 32, 34a, 36–40, 42, 47a, 54a, 65–68), and subsequently corrected using CorelDRAW Graphics Suite X6.

The material examined for morphological study and measurements is listed in Tables 1 and 3. Chaetotaxy nomenclature, symbols, and abbreviations follow Ashe and Watrous (1984), and the morphological description style is according to Staniec et al. (2016). The voucher specimens are deposited in the collection of the Department of Zoology, Marie Curie-Sklodowska University, Lublin.

## Results

### Generic diagnosis of the mature larvae

The combination of characteristics that enable mature larvae of *Dinaraea* to be distinguished from known larvae of other genera within the subfamily Aleocharinae are as follows (Paulian 1941, Pototskaya 1967, Topp 1975, Ashe 1981, 1985, Ashe and Watrous 1984, Ahn 1997, Jeon and Ahn 2009, Staniec et al. 2009, 2010, 2016, Zagaja et al. 2014, the present study): (1) body narrow, elongate, dorso-ventrally flattened, sides almost parallel; (2) pronotum slightly wider than (at most 1.1 as wide as) head; (3) antennal article I longer than wide; (4) sensory appendage (Sa) of antennal article II acorn-shaped, longer than antennal article III; (5) length ratio of antennal articles I and II – 1:1.6; (6) central region of anterior margin of labrum protruding and crenate; (7) mandibles with one large and two-five small subapical teeth; (8) mala at least slightly widened at adoral margin; (9) adoral margin of mala with eight large and approx. 15 small teeth; (10) length ratio of article I and III of maxillary palp – 1:1.5; (11) ligula finger-like, 2.5 × as long as wide; (12) hypopharynx with approx. 60 triangular microtrichia directed towards the central area without microtrichia; (13) length ratio of articles I and II of labial palp – 1:2.1; (14) pronotum without seta Da1; (15) on abdominal sternite I seta P5 present, seta D3 absent; (16) abdominal segment X approx. 2.5 × as long as urogomphus (without apical seta); (17) length ratio of urogomphus to its apical seta – 1:1.6.

### Description of larval stages (*D. aequata*-*D.a.*; *D. linearis*-*D.l.*)

**Figures 1–11.**
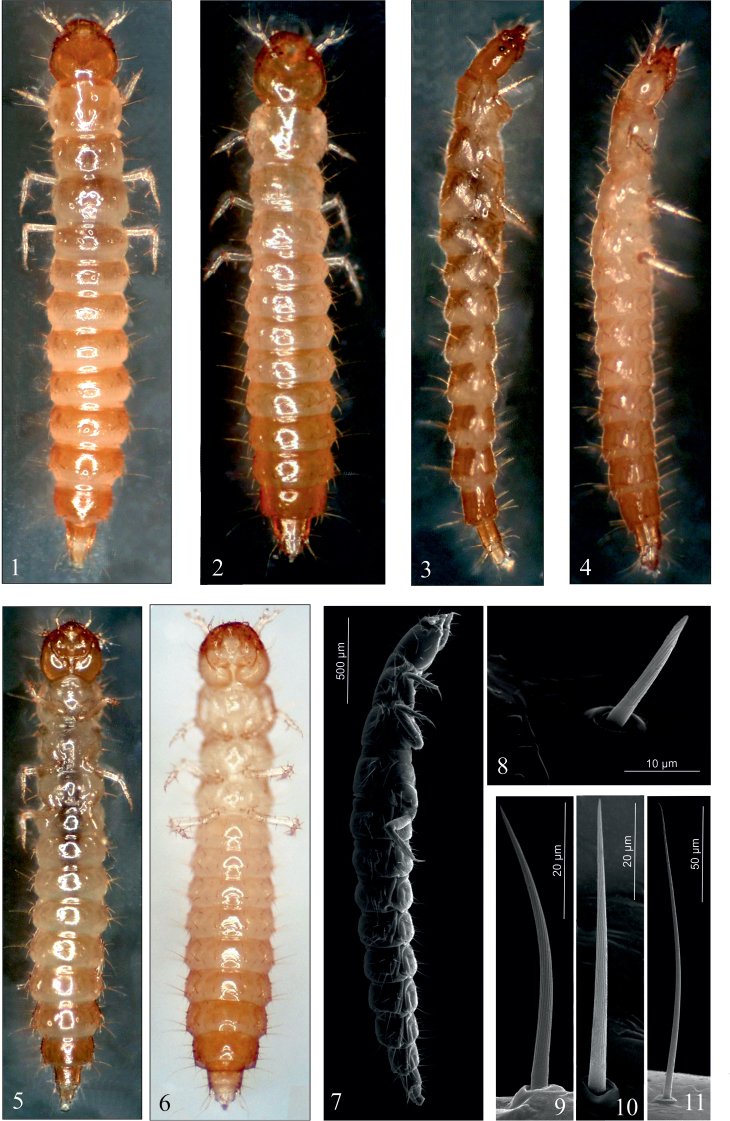
*D. aequata* (**1**, **3**, **5**, **7**–**11**), *D. linearis* (**2**, **4**, **6**), mature larva. **1–7** habitus in dorsal (**1**, **2**), lateral (**3**, **4**, **7**) and ventral (**5**, **6**) aspect, **8–11** setae near epicranial suture (**8**) of abdominal tergites (**9–11**).

***Late larval instars (L2–3)* (Figs 1–7)**

Body narrow, elongate, semi-cylindrical, distinctly (*D.a.*) or moderately (*D.l.*) dorso-ventrally flattened, sides almost parallel, head slightly narrower than prothorax and as wide as mesothorax, pro- and metathorax almost equal in width, abdomen gradually widening to segments IV or V, then tapering to terminal segment of body; segments IX and X distinctly narrower than the others. Colour: whole head reddish brown (*D.a.*) or anterior area of head reddish brown, but posterior distinctly paler (*D.l.*), ocellus dark; all tergites yellowish brown (*D.a.*) or all thoracic and abdominal tergites I–V almost colourless, then tergites gradually darkening from yellow (VI) to yellowish brown (VII) and reddish brown (VIII, IX) *(D.l.)*; abdominal sternites gradually darkening from yellowish brown (I) to brown (VIII and IX) (*D.a.*) or abdominal sternites I–V almost colourless but the others somewhat darker (*D. l.*); legs and abdominal segment X colourless. Setae of different length, light brown, simple with longitudinal grooves (Figs 8–11). Microstructure of head and tergites as in Figs 15b, 54a.

**Figures 12–23.**
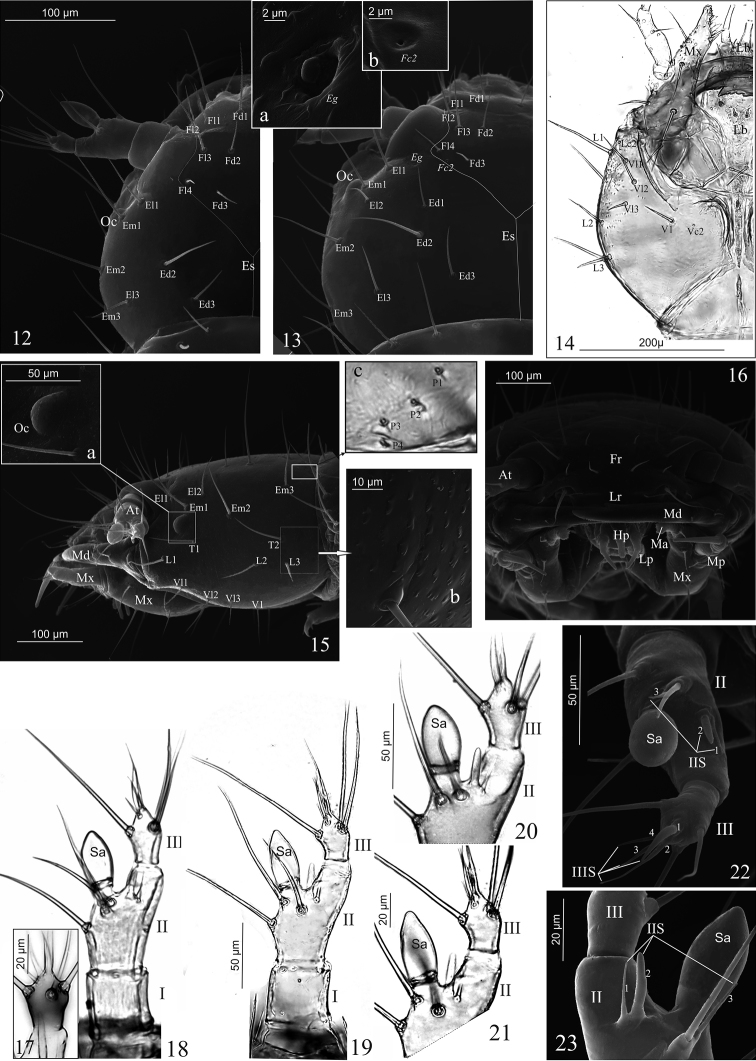
*D. aequata* (**12**, **13**, **15**, **16–18**, **20**, **22**, **23**) *D. linearis* (**14**, **19**, **21**). First larval instar (**12**, **17**), mature larva (**13–16**, **18–23**), **12–16** head in dorsal (**12**, **13**), ventral (**14**), lateral (**15**) and frontal (**16**) aspect with glands (**13**a, **13**b), ocellus (**15a**), microstructure (**15b**) and posterior setae (**15c**), **17–23** right antenna, article III in dorsal aspect (**17**), entire in dorsal aspect (**18**, **19**), anterior region in dorsal aspect (**20**, **21**), entire in apical aspect (**22**), anterior region of article II in ventral aspect (**23**) Abbreviations: **I–III** antennal articles, **IIS IIIS** solenidia of antennal article II or III, **At** antenna, **Ed** epicranial dorsal setae, **Eg** epicranial gland, **El** epicranial lateral setae, **Em** epicranial marginal setae, **Es** epicranial suture, **Fc** frontal campaniform sensilla, **Fd** frontal dorsal setae, **Fl** frontal lateral setae, **F** frons, **Hp** hypopharynx, **L** lateral setae, **Lb** labium, **Lc** lateral campaniform sensilla, **Lp** labial palp, **Lr** labrum, **Ma** mala, **Md** mandible, **Mx** maxilla, **Mp** maxillary palp, **Oc** ocellus, **P** posterior setae, **Pl** labial palp, **Pm** maxillary palp, **Sa** sensory appendage, **T** temporal setae, **V** ventral setae, **Vc** ventral campaniform sensilla, **Vl** ventral lateral setae.

Head (Figs 13–16): almost as long as wide, widest at level of setae Ed3, lateral margins distinctly rounded; dorsal ecdysial lines (Es) bifurcate at approx. half the head length; each side of head with one oval, weakly convex, black ocellus (Oc) (Figs 3, 4, 12, 13, 15, 15a). Chaetotaxy of dorsal side with 40 setae – 14 frontal [2(Fd1–3, Fl1–4)], 18 epicranial [2(Ed1–3, Ell-3, Em1-3)], eight posterior micro setae (2P1–4); a pair of frontal campaniform sensillae (Fc2) and epicranial glands (Eg) (Figs 13, 13a, 13b, 15c). Lateral margins with ten setae [2(T1–2, L1–3)] (Fig. 15). Ventral side with eight setae [2(Vl1–3, V1)], and a pair of ventral (Vc2) and lateral (Lc2) campaniform sensillae. Functional position of antennae (At), labrum (Lr), mandibles (Md), maxillae (Mx), hypopharynx (Hp), and labium (Lb) as in Figs 14–16.

Antenna (Figs 18–21): three-articled, length ratio of articles I–III: 1.0:1.9:1.2 (*D.a.*) or 1.0:2.4:1.4 (*D.l.*). Article I almost 1.0–1.1 × as long as wide, with four pores; article II 1.8 × as long as wide, with three macro setae, one acorn-shaped sensory appendage (Sa), 1.8 (*D.a.*) or 2.1 (*D.l.*) × as long as wide (Figs 20, 21), and three solenidia ventrally of different size (IIS1–3) (Figs 22, 23); Sa longer than article III; article III 1.3–1.4 × as long as wide, with three macro setae and four solenidia apically (IIIS1–4) of different length (Fig. 22).

**Figures 24–32.**
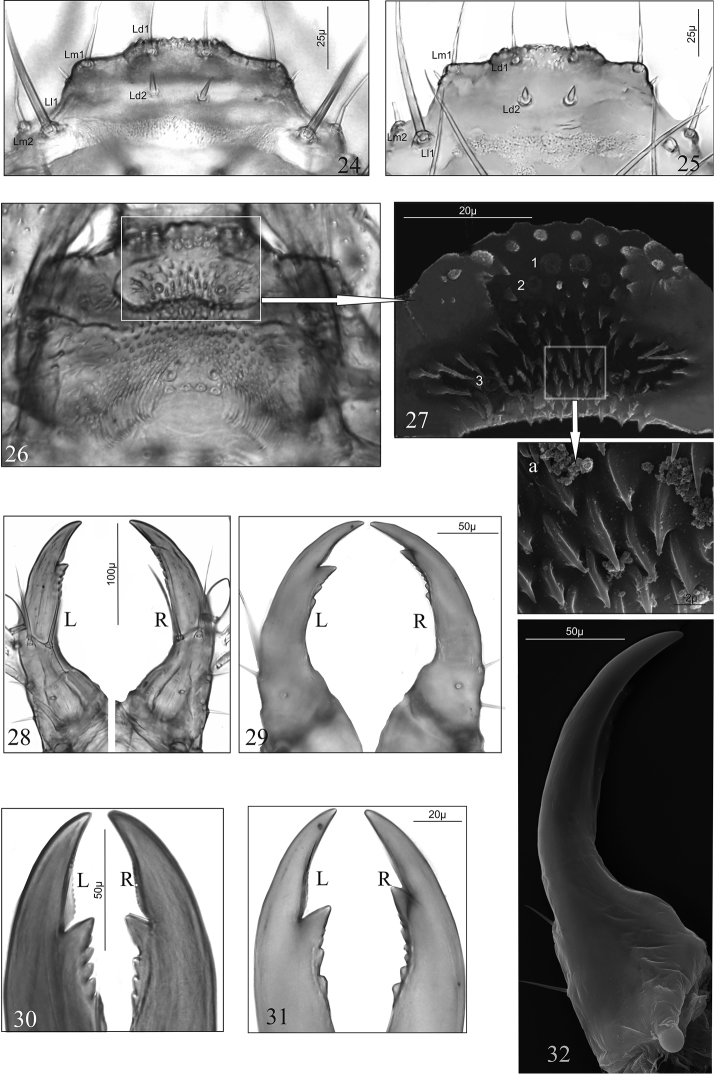
*D. aequata* (**24**, **26**, **27**, **28**, **30**, **32**) *D. linearis* (**25**, **29**, **31**), mature larva. **24**, **25** labrum **26**, **27**, **27a** epipharynx, **28**, **29** left (**L**) and right (**R**) mandible in dorsal aspect **30**, **31** anterior region of left (**L**) and right (**R**) mandible in dorsal aspect **32** right mandible in ventral aspect. Abbreviations: **Ld** labral dorsal setae, **Lm** labral marginal setae, **Ll** labral lateral setae.

Labrum (Figs 24, 25): trapeziform in outline, central region of anterior margin protruding and crenate, length ratio of protruding region and whole anterior margin 1:2 (*D.a.*) or 1:1.7 (*D.l.*); with eight macro [2(Ld1, Lm1, Lm2, Ll1)] and two micro, spine-shaped (Ld2) setae; separated from clypeal region by membranous area. Adoral surface of labrum (epipharynx) (Figs 26, 27): membranous with numerous, pointed cuticular processes directed to central area of epipharynx (Fig. 27a) and three pairs of pores (coded: 1–3; Fig. 27).

Mandibles (Md) (Figs 28, 29, 32): elongate, strongly bent, moderately widened basally, with two macro setae near the outer margin and a pore; incisor lobe with one large and a different number (from two to five) of small subapical teeth: four teeth in left (L) and three teeth in right (R) mandible (*D.a.*) or two teeth in left (L) and five teeth in right (R) mandible (*D.l.*) (Figs 30, 31).

**Figures 33–40.**
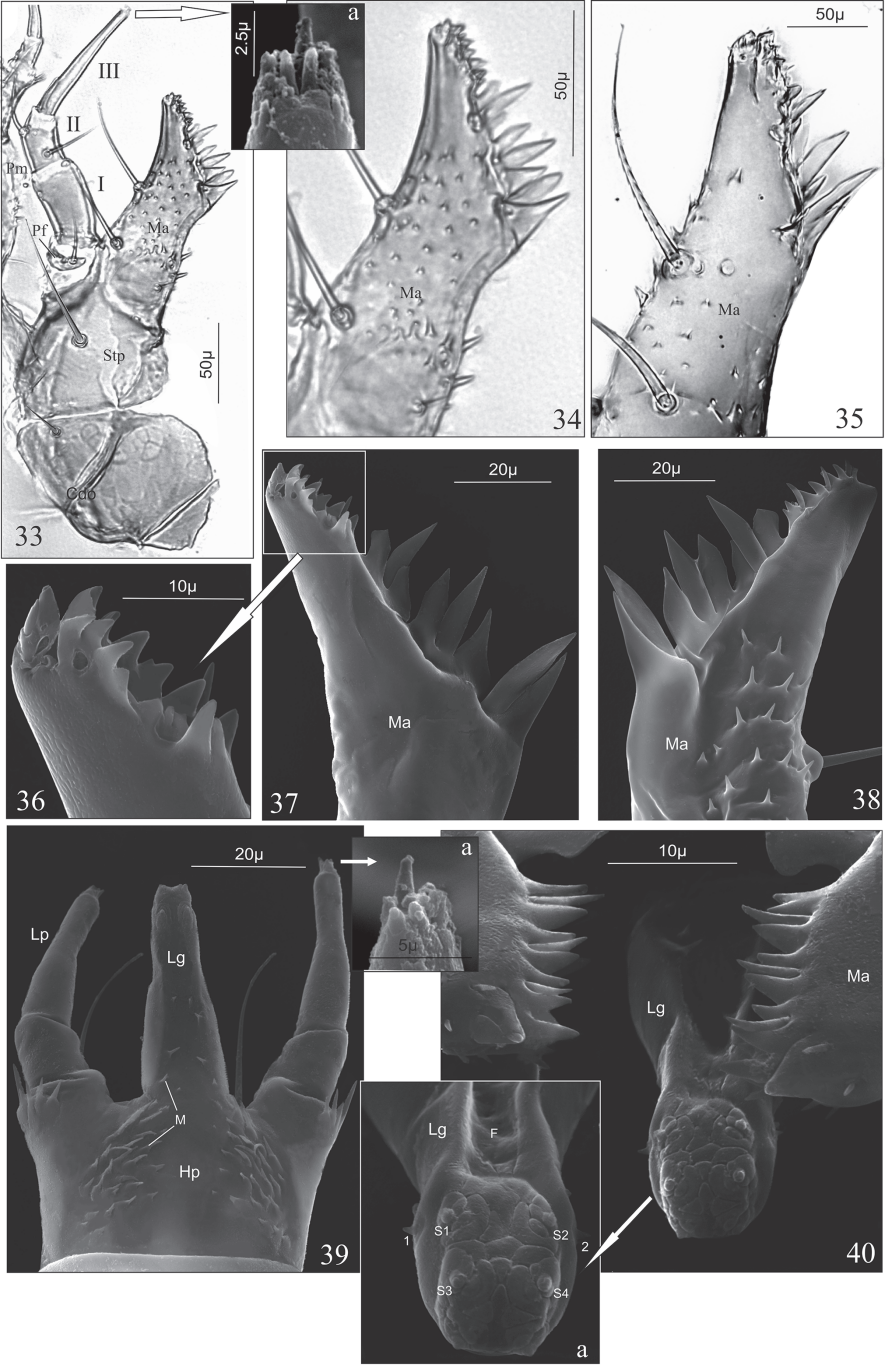
*D. aequata* (**33**, **34**, **36–40**) *D. linearis* (**35**), mature larva. **33** right maxilla in ventral aspect and apex of maxillary palp (**33a**), **34**, **35** right mala in ventral aspect **36–38** adoral margin of left mala in dorsal (**36**, **37**) and ventral (**38**) aspect **39**, **39a** labium and apex of labial palp, view from hypopharynx, **40** functional position of hypopharynx and adoral margins of malae and apex of ligula (**40a**). Abbreviations: **I–III** articles of maxillary palp, **Cd** cardo, **F** furrow, **Hp** hypopharynx, **Lg** ligula, **Lp** labial palp, **M** microtrichia, **Ma** mala, **Pf** palpifer, **Pm** maxillary palp, **1–2** and **S1–4** sensilla, **Stp** stipes.

Maxilla (Mx) (Fig. 33): consisting of triangular cardo (Cd) divided by sclerotised ridge into two unequal parts, shorted stipes (Stp), slender, obliquely truncate mala (Ma) distinctly (*D.a.*) or slightly (*D.l.*) widened at adoral margin (Figs 34, 35), palpifer (Pf) and three articled maxillary palp (Pm); cardo with one ventral seta; stipes with two setae; palpifer with one seta; mala separated from stipes by clearly visible line, with two setae, one pore and approx. 40 (*D.a.*) or 25 (*D.l.*) triangular cuticular processes ventrally; adoral margin of mala (functional positions in Fig. 40) with group of approx. 15 micro teeth apically (Fig. 36) and ctenidium of eight macro teeth, dagger-shaped, different sizes (Figs 37, 38).

Maxillary palp (Pm) (Fig. 33): length ratio of articles I–III: 1.6:1:2.2; article I wider than second, 1.8 × as long as wide with two pores; article II 1.5 × as long as wide with two setae; article III narrower than I and II, tapering slightly to apex, 6.8 × as long as wide, with one digitiform sensory appendage basally 0.3 × as long as article, one pore and a few tiny sensory appendages apically, among them the central one higher than the others (Fig. 33a).

Hypopharynx (Hp) (Fig. 39): membranous, surface (except central area) with approx. 60 triangular microtrichiae (M) directed to the central area without microtrichiae. Ligula (Lg) (Figs 39, 40): elongate, finger-like, gradually tapering to the top, approx. 2.5 × as long as wide at the base, with deep longitudinal furrow (F) and a few microtrichia laterodorsally; distal part with two spinose (coded: 1–2) and four button-like (coded: S1–4) sensilla, surface of apex with microsculpture resembling dermatoglyphics (Fig. 40a). Prementum (Pmnt) trapeziform, 1.4 × as wide at the base as long, with two long setae and four cuticular processes at the base of each labial palp (Lp) (Figs 14, 39). Labial palp two-articled, length ratio of articles I and II: 1:2.1, article I 1.1 × as long as wide, article II 3.5 × as long as wide with a few sensory appendages apically, among them the central one higher than the others (Fig. 39a).

**Figures 41–48.**
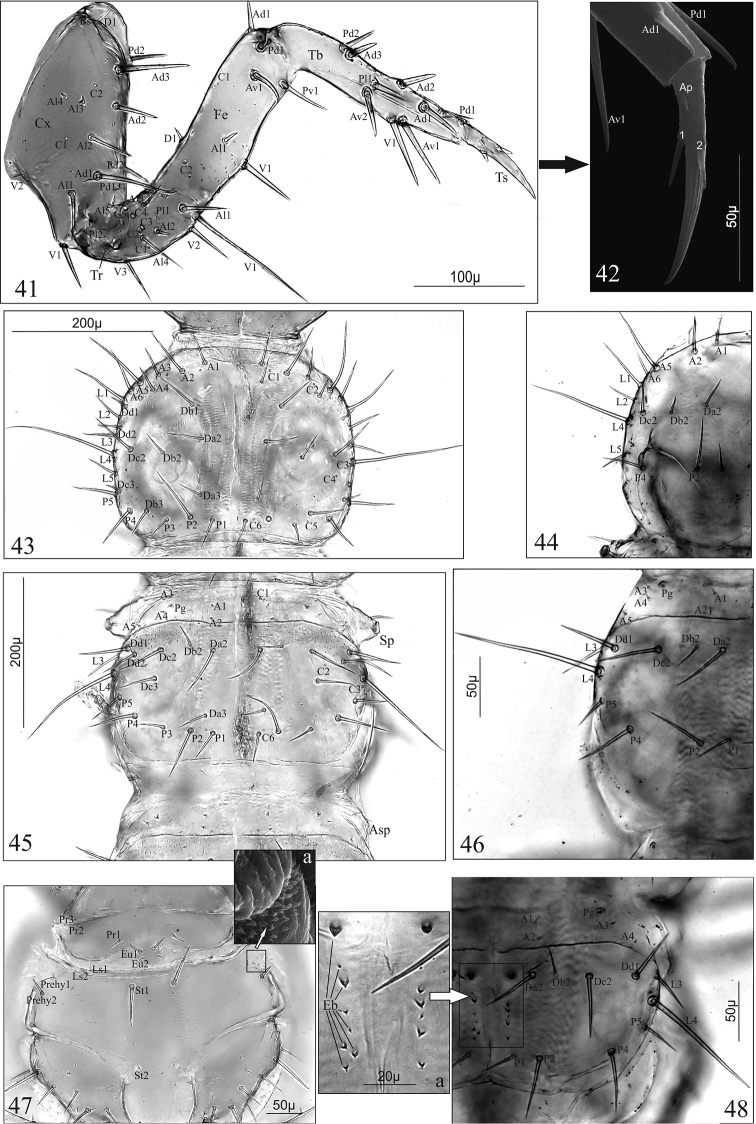
*D. linearis*, mature larva (**41**, **42**, **43**, **45**, **47**, **47a**), first larval instar (**44**, **46**, **48**, **48a**). **41**, **42** fore right leg in anterior aspect and tarsungulus (**42**), **43**, **44** pronotum, **45**, **46** mesonotum, **47** prosternum with microstructure (**47a**), **48** metanotum with egg-bursters (**48a**). Abbreviations: **A** anterior setae, **Ad** anterodorsal setae, **Al** anterolateral setae, **Ap** appendage, **Asp** atrophied spiracles, **Av** anteroventral setae, **Bs** basal setae, **C** campaniform sensilla, **Cx** coxa, **Eb** egg-bursters, **Eu** eusternum, **Fe** femur, **Da–d** dorsal setae, **L** lateral setae, **Ls** laterosternum, **P** posterior setae, **Pd** posterodorsal setae, **Pg** pretergal gland, **Pr** presternum, **Prehy** prehypopleuron, **Pv** posteroventral setae, **Sp** spiracle, **St** sternellum, **Tb** tibia, **Tr** trochanter, **Ts** tarsungulus, **V** ventral setae.

Thorax. Foreleg (Fig. 41): consists of stocky coxa (Cx), short trochanter (Tr), elongated femur (Fe) 3 × as long as wide, slim tibia (Tb) 5.3 × as long as wide and tarsungulus (Ts) slightly curving inwards, 7.1 × as long as wide; Cx with 13 setae (Ad1–3, Al1–4, Bs, D1, Pd1–2, V1–2) and two pores (C1–2); Tr with 10 setae (Al1–5, Pl1v2, V1v3) and 5 pores (C1–5); Fe with 7 setae (Ad1, Av1, Al1, D1, Pd1, Pv1, V1) and two pores (C1–2); Tb with nine spine-shaped setae (Ad1–3, Av1–2, Pd1–2, Pl1, V1); Ts with two spine-shaped setae and one appendage (Ap) (Figs 41, 42). Length ratio of Fe, Tb and Ts: 1.9:2.2:1. Length ratio of pronotum (Pnt), mesonotum (Msn) and metanotum (Mtn): 1.4:1:1.3. Pnt with 50 setae [2(A1–6, Da2–3, Db1–3, Dc2–3, Dd1–2, L1–5, P1–4)] and 12 pores (2[C1–6]) (Fig. 43); Msn with 38 setae [2(A1–5, Da2–3, Db2, Dc2–3, Dd1–2, L3–4, P1–5)] and eight pores [(2C1, C2, C3, C6)]; chaetotaxy of metanotum identical with that of mesonotum; lateral area between pro- and mesothorax with a pair of functional spiracles (Sp), and between meso- and metathorax with a pair of atrophied spiracles (Asp) and one micro seta (Fig. 45). Prosternum (Fig. 47) with 22 setae [2(Eu1–2, Ls1–2, Pr1–3, Prehy1–2, St1–2)] and microstructure laterally (Fig. 47a).

**Figures 49–53.**
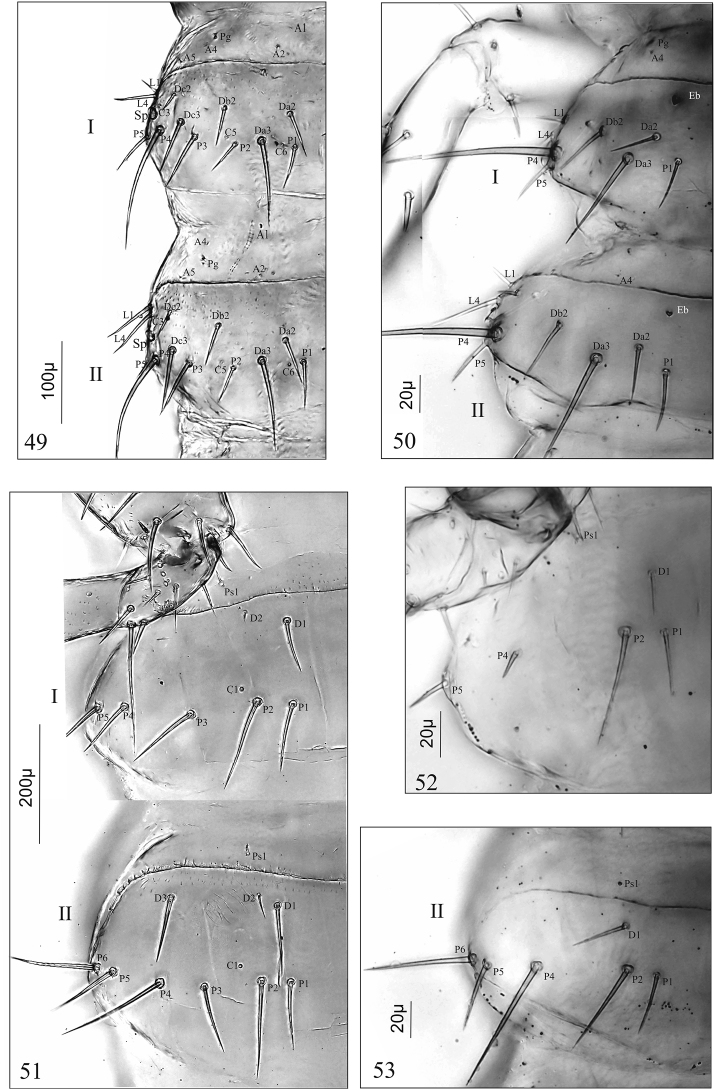
*
D. aequata*, mature larva (**49**, **51**), first larval instar (**50**, **52**, **53**). **49**, **50** abdominal tergites I and II **51** abdominal sternites I and II **52** abdominal sternites I **53** abdominal sternites II. Abbreviations: **A** anterior setae, **C** campaniform sensilla, **D**, **Da–c** discal setae, **Eb** egg-bursters, **L** lateral setae, **P** posterior setae, **Pg** pretergal gland, **Ps** presternal setae, **Sp** spiracle.

**Figures 54–68.**
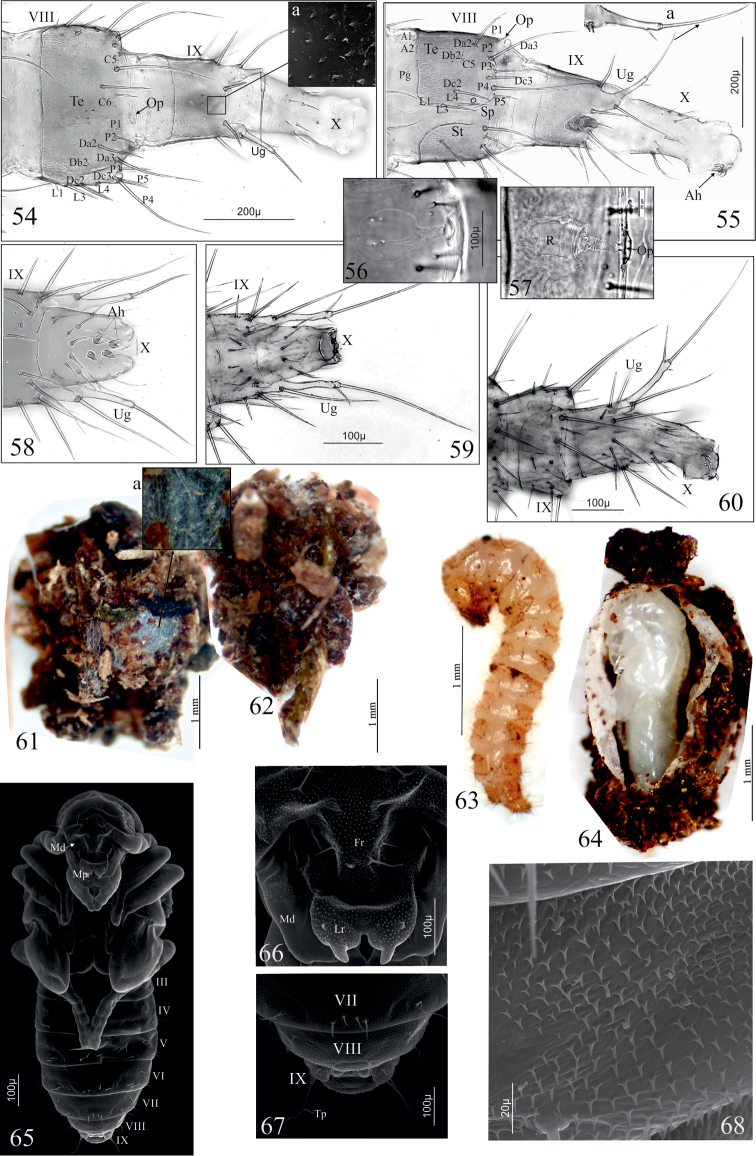
*D. linearis* (**54–57, 60–64**), *D. aequata* (**58, 59, 65–68**), mature larva (**54–56, 58**), first larval instar (**57, 59, 60**), prepupa (**61**), cocoon (**62**, **63**), cocoon and pupa (**64**), pupa (**65–68**). **54, 55** abdominal segments VIII-X in dorsal (**54**) and lateral (**55**) aspect **56**, **57** tergal gland reservoir of segment VIII **58–60** abdominal segments IX and X in ventral (**58, 59**) and lateral (**60**) aspect **61, 62** pupal cocoon **63** prepupa in lateral aspect **64** ripped cocoon with pupa inside in lateral aspect **65–68** pupa in ventral aspect (**65**), front of head (**66**), terminal abdominal sternites VII–IX (**67**) and microstructure of abdominal sternite VIII (**68**). Abbreviations: **Ah** anal hooks, **Fr** frons, **Lr** labrum, **Md** mandible, **Mp** maxillary palp, **Op** opening of gland reservoir, **Pg** pretergal gland, **R** tergal gland reservoir, **Sp** spiracle, **St** sternite, **Te** tergite, **Tp** terminal prolongation, **Ug** urogomphus.

Abdomen. Chaetotaxy of tergites: I–VII with 32 setae [2(A1–2, A4–5, Da2–3, Db2, Dc2–3, L1, L4, P1–5), six pores [2(C3, C5, C6)] and a pair of glands (Pg) (Fig. 49); VIII with 30 setae [2(A1–2, Da2–3, Db2, Dc2–3, L1, L3–4, P1–5)], two pores (C5) and a pair of glands (Pg) (Figs 54, 55). Tergal gland reservoir (R) clearly developed with split opening (Op) at the posterior margin of abdominal tergite VIII (Figs 54–56). Chaetotaxy of sternites: I (Fig. 51) with 16 setae (2[D1–2, Ps1, P1–5]); II–VIII (Fig. 51) with 20 setae (2[D1–3, Ps1, P1–6]). Segment IX and X with tergites and sternites fused in uniform ring; segment IX with 28 setae (six micro) (Figs 54, 55). Urogomphi (Ug) of segment IX (Figs 54, 55): two-articled, article I fused to tergum IX; article II slender, finger-shaped, moderately tapering apically, 1.5 × as long as basal article, 4.1 × as long as wide, with one short seta subapically, one macro seta apically and a pore basally; length ratio of Ug and apical seta: 1:1; length ratio of urogomphus (without apical seta) and segment X (pygopod): 1:1.5. Segment X with 16 setae and four anal hooks (Ah) (Fig. 58).

***First larval instar* (*L_1_*) (Figs 13, 17, 44, 46, 48, 48a, 50, 52, 53, 57, 59, 60)**

The main differences between L1 and L2–L3 of *Dinaraea* involve: (1) colouration of last (III) antennal article: brown in L1, almost colourless in L2–L3; (2) chaetotaxy of head, pro-, meso-, metanotum, abdominal tergites and sternites I–VII: fewer setae in L1 than in L2–3; (3) egg bursters on metanotum (a pair of large ones) and abdominal segments I–III: present in L1, absent in L2–L3; (4) size of gland reservoir of segment VIII; (5) shape of urogomphi: slightly inward-curving in L1, straight in L2–L3; (10) number and length of subapical and apical setae of urogomphi. Some differences in measurements of all larval instars are shown in Table 1. For more details regarding the differences between L1 and L2–L3, see Table 2.

Pupal cocoon (Figs 61, 61a, 62, 64); before pupation, the mature larva (L3) spins a silken cocoon into which it weaves particles of the surrounding substrate; length 3.0 mm, width 1.8 mm. Prepupa as in Fig. 63.

**Table 1.** Some measurements of larval instars of *Dinaraea aequata* and *D. linearis*. Symbols and abbreviations: L1–L3 larval instars, A average, N number of specimens, R range, SV standard variation.

**Character**	**Species**	**L1**		**L2**		**L3**	
		A/R	N/SV	A/R	N/SV	A/R	N/SV
Body length	*D. aequata*	1.93/1.61–2.21	7/0.21	2.31/1.61–2.83	5/0.46	3.42/3.01–3.78	11/0.24
	*D. linearis*	1.6/1.41–1.89	6/0.18	2.23/1.92–2.48	5/0.27	2.79/2.23–3.12	12/0.25
Epicranium length	*D. aequata*	0.26/0.24–0.28	7/0.01	0.34/0.34–0.35	5/0.01	0.43/0.42–0.46	11/0.01
	*D. linearis*	0.24/0.22–0.27	6/0.01	0.31/0.28–0.34	5/0.02	0.36/0.34–0.39	11/0.02
Epicranium width	*D. aequata*	0.28/0.28–0.28	7/0.00	0.35/0.34–0.35	5/0.01	0.43/0.42–0.45	11/0.01
	*D. linearis*	0.25/0.25–0.25	6/0.00	0.31/0.29–0.32	5/0.01	0.37/0.35–0.38	12/0.01
Pronotum width	*D. aequata*	0.29/0.28–0.29	7/0.01	0.36/0.34–0.36	5/0.01	0.49/0.46–0.50	11/0.01
	*D. linearis*	0.27/0.25–0.28	6/0.01	0.34/0.32–0.36	5/0.02	0.43/0.41–0.52	12/0.03

**Table 2.** Some differences in chaetotaxy between early (L1) and late (L2–3) larval instars of *Dinaraea*, *Thiasophila*, and *Gyrophaena*. Abbreviations: Ar article, As apical seta of urogomphus, At antenna, D dorsal, Ep epicranial part, l long, Ls lateral margin, Lr length ratio, Msn mesonotum, Mtn metanotum, NrS number of setae, NrEb number of egg bursters, Pnt pronotum, S segment, s short, Sas subapical setae of urogomphus, St sternite, Te tergite, Ug urogomphus, (…) new setae, ? no data available. Data based on Zagaja et al. (2014), Staniec et al. (2016), and present study.

**Characters**	**Athetini**		**Oxypodini**		**Homalotini**	
	***Dinaraea* (*D. aequata*, *D. linearis*)**		***Thiasophila* (*T. angulata*)**		***Gyrophaena* (*G. boleti*)**	
**Head**						
	L1	L2–3	L1	L2–3	L1	L2–3
NrS: Ep	14	18 (Ed1, El2)	14	18 (Ea1, Ed1)	12	12
At: Ar III	dark	light	dark	light	dark	light
**Thorax**						
NrS: Pnt	28	50 2(A3, A4, Da3, Db2, Db3, Dc3, Dd1, Dd1, L3, P3, P5)	28	52 2(A3, Da1, Da3, Db1, Db3, Dc1, Dc3, Dd1, L2–3, L5, P3)	22	30 2(A3, A5, P3, L5)
NrS: Msn, Mtn, each	30	38 2(Da3, Dc3, Dd2, P3)	30	38 2(Da3, Db3, Dc2, P3)	16	18 2P3
NrEb: Msn	lack	lack	lack	lack	two	lack
NrEb: Mtn	14 (2big)	lack	approx. ten	lack (six big)	nine	lack
**Abdomen**						
NrEb: Te I–II/each	two	lack	two	lack	two	lack
NrEb: Te III–IV/each	lack	lack	lack	lack	two	lack
NrS: Te I–VIII	18	32 2(A1, A2, A5, P2, P3, Dc3, Dc2)	24	30 2(Db3, Dc2, P3)	14	18 2(Db3, P3)
NrS: St I	12	16 2(D2, P3)	ten	14 2(D2, Ps1)	ten	ten
NrS: St II–VII	14	20 2(D2, D3, P3)	14	20 2(D2, D3, P3)	12	16 2(D2, P3)
NrS: St VIII	14	20 2(D2, D3, P3)	14	20 2(D2, D3, P3)	12	14 2(D2)
Ug: Sas	2 /s and l/	1 s	2 /s and l/	1 s	2 /s and l/	1 s
Lr Ug to As	1:1.6	1:1	1:2.3	1.1:1	1:2.2	1:1.1
Lr Ug to S X	1:1.1	1:1.5	1.1:1	1:1.5	1.2:1	1:1

### Morphological comments on the *Dinaraea pupa* (based on *D. aequata*)

Because of the poor state of preservation of most of the reared research material, this description covers only the ventral part of the female pupa.

Pupa (Figs 64–68). Some measurements of pupae of both species are listed in Table 3. Pupa exarate, body moderately flattened dorso-ventrally, slightly sclerotised, with numerous setae growing from basal, cuticular protuberances (Fig. 66); colour white, long setae pale brown, short setae almost colourless. Head: directed downwards with 24 setae (among them two very short ones on labrum, four small ones at base of labrum) (Fig. 65). Labrum: anterior margin with deep incision dividing labrum into two parts and a pair of finger-like protuberances. Maxillary palp long, protruding beyond half-length of fore tarsi. Antennae: curved, lying on the fore and middle knees, protruding distinctly beyond apex of middle of knees (Fig. 65). Hind tarsi almost reaching middle of visible abdominal sternite III (actually V). Setae of abdominal sternites relatively short, numbers of setae on sternites: IV – 8, V–VII – each with 14, VIII, IX – each with eight setae (Figs 65, 67). Last tergite IX extended into two abdominal processes, each with one relatively long terminal prolongation (Tp) (Fig. 67). Terminal segments (IX) in female with double gonotheca as in Fig. 67. Microstructure of abdominal sclerites as in Fig. 68.

**Table 3.** Some measurements of pupae of *Dinaraea aequata* and *D. linearis*. Abbreviations: A average, N number of specimens, R range, SV standard variation.

**Species**	**Body length**		**Body width**		**Epicranium width**		**Pronotum width**	
	A/R	N/SV	A/R	N/SV	A/R	N/SV	A/R	N/SV
*D. aequata*	2.48/2.42–2.53	3/0.08	1.12/1.08–1.15	3/0.05	0.53/0.52–0.54	3/0.01	0.63/0.62–0.64	3/0,01
*D. linearis*	2.16/2.13–2.20	4/0.03	0.87/0.7–1.0	4/0.26	0.49/0.49–0.49	4/0.0	0.57/0.54–0.59	4/0.02

**Table 4.** Morphological differences between mature larva (L3) of *Dinaraea aequata* and *D. linearis*. Abbreviations: Lb labrum, Lr length ratio, LWr length to width ratio, MdL MdR left (L) and right (R) mandible, Nr number, Sa sensory appendage of antennal article II, NrSt number of subapical teeth.

**Character**	***D. aequata***	***D. linearis***	**Figures**
**Body – colour/appearance**			
Head: reddish brown	whole	posterior area distinctly lighter	1–6
Tergites	all yellowish brown	thoracic and abdominal I–V colourless	1–4
Abdominal sternites I–V	yellowish brown	almost colourless	5, 6
Dorso-ventrally flattened	distinctly	moderately	3, 4
**Antenna**			
Lr of articles I–III	1.0:1.9:1.2, respectively	1.0:2.4:1.4, respectively	18, 19
Sa: LWr	1.8:1	2.1:1	20,21
**Mouthparts**			
Lb: Lr of protruding region and whole anterior margin	1:2	1:1.7	24, 25
Lb: setae of Ld2	moderately elongate	extremely shortened	24, 25
Left mandible: NrSt	one big + four small	one big + two small well visible	30, 31
Right mandible: NrSt	one big + three small	one big + five small well visible	30, 31
Ma: widened at adoral margin	distinctly	slightly	34, 35
Ma: Nr of cuticular processes	approx. 40	approx. 25	34, 35

### Notes on distribution, ecological preferences, and life history of *D. aequata* and *D. linearis*

***Distribution***

The geographical distributions of *D. aequata* and *D. linearis* are very similar. Both species are known from the majority of European countries, the Asian part of Russia (Siberia, Far East) and northern China. In Poland, they probably occur all over the country, although the former is usually come across more often than the latter (Burakowski et al. 1981, Löbl and Löbl 2015, unpublished data).

***Ecological preferences***

*Dinaraea aequata* and *D. linearis* have similar ecological preferences. Being saproxylic species, they are associated with damp, rotting tree trunks and stumps, mainly of deciduous trees, including *Acer*, *Alnus*, *Betula*, *Fagus*, *Fraxinus*, *Populus*, and *Quercus*. Koch (1989) has described them as eurytopic and corticolous species. They can be found in various types of woodland and other groups of trees, under loose bark, in the corridors of bark beetle larvae, in the rotten wood itself, or, especially the former species, in various polypore species (from the genera *Bjerkandera*, *Fomitopsis*, *Fomes*, *Funalia*, *Ganoderma*, *Inonotus*, *Piptoporus*, *Polyporus*, *Trametes*, etc.) (Nikitsky and Schigel 2004, authors’ observations).

Recent research into saproxylic beetles in several regions of Poland (the Wielkopolska Plain, the Bieszczady Mts., the Eastern and Western Beskid Mts., the Pieniny Mts., the Lublin Upland), has yielded a range of fresh information regarding the environmental preferences of these two *Dinaraea* species. In the lowlands, *D. aequata* was found in May and December under the bark of a standing birch (*Betula* L.) and the wet bark of a wind-thrown aspen trunk (*Populus tremula* L.), together with the following other species of beetles: *Anomognathus cuspidatus*, *Batrisodes venustus*, *Bolitochara obliqua*, *Dinaraea angustula*, *Hololepta plana*, and *Siagonium quadricorne*. In mountain and piedmont regions this species was captured from June to July and in November–December under the bark of the following broad-leaved tree species: beech (*Fagus sylvatica* L.), sycamore (*Acer pseudoplatanus* L.), field maple (*Acer campestre* L.), a burnt bird cherry (*Padus avium* Mill.), and the remains of firs (*Abies alba* Mill.), together with *Bolitochara obliqua*, *Corticeus unicolor*, *Homalota plana*, *Ipidia binotata*, *Phloeocharis subtilissima*, *Pissodes piceae*, *Xylostiba monilicornis*, and *Ropalopus macropus*. Some of *D. aequata* beetles have also been recorded on fungi *Auricularia auricula-judae* (Bull.) Quél. and *Sarcodontia crocea* (Schwein.) Kotl., along with *Acrulia inflata*, *Atheta crassicornis*, *Atheta ravilla*, *Autalia longicornis*, *Bolitochara obliqua*, *Gyrophaena manca*, *Oxypoda alternans*, and *Scaphisoma boleti*. In Poland *D. linearis* appears to be rarer than *D. aequata*. It has been recorded in January, March, July, and August, exclusively under the bark of various trees. In the lowlands these were larch (*Larix* Mill.), pine (*Pinus* L.), oak (*Quercus* L.), a wind-thrown ash (*Fraxinus* L.), the mountains lime (*Tilia* L.), and fir. Accompanying beetles included *Corticeus unicolor*, *Gasterocercus depressirostris*, *Paromalus parallelepipedus*, *Phloeostiba lapponica*, *Rhyncolus elongatus*, *Scaphisoma agaricinum*, *Silvanus bidentatus*, and *Tetropium gabrieli* (Melke, oral information).

***Notes on the life history in the laboratory***

In the rearing of adults of *D. aequata* (temp. 20 °C±3), started on 19 November 2004, the first larvae (L1 and L2) were observed just six days later (25 November), and eight and ten days later the first prepupae and pupae respectively appeared. Various late developmental stages (mostly L3 and pupae) were observed until 20 January 2005. Larval development in the rearing of adults of *D. linearis*, started on 6 December 2004, was observed from 1 January to 11 February, and pupation from 19 to 21 February 2005. The larval and imaginal forms of both species were fed exclusively with springtails from the family *Onychiuridae*. On several occasions foraging larvae of *D. aequata* were observed as they caught their victims of various sizes in their mandibles. Within the following 5 to 10 minutes they consumed most of the springtail bodies, but always rejected fragments of the carapace.

## Discussion and summary

This paper describes in detail the external morphology of the hitherto unknown larval stage of the genus *Dinaraea*, including the chaetotaxy, using the terminology proposed for the subfamily Aleocharinae by Ashe and Watrous (1984). The description is based on individuals from European populations of *D. aequata* and *D. linearis*, the larvae of which were bred in the laboratory from imagines. The morphological differences between the mature larvae of the two species relate to: (a) the dimensions of various body parts, they are larger in *D. aequata* (Table 1); (b) colour of head, thoracic tergites and anterior abdominal tergites and sternites, generally darker in *D. aequata*; (c) degree of body flattening, somewhat more flattened in *D. aequata*; (d) shape of article II of antenna and Sa of article III, slightly more elongate in *D. linearis*; (e) structural details of the anterior margin of the labrum and the length of its setae Ld2; (f) number of subapical teeth on both mandibles, more on the left-hand one in *D. aequata*, more on the right-hand one in *D. linearis*; (g) shape of mala, more protracted at adoral margin in *D. aequata*; (h) number of cuticular processes on mala, more in *D. aequata* (Table 4). These diagnostic features distinguish the larvae of these two species, which may co-occur under natural conditions. Particularly useful in this respect are features a–c, relating to the dimensions of the body and its general appearance. Very many more features distinguishing aleocharine larvae belonging to the same genus were established for *Haploglossa* Kraatz, 1865 (Staniec et al. 2010). A comparative analysis of *H. picipennis* (Gyllenhal, 1827) and *H. nidicola* (Fairmaire, 1852) revealed differences not only in larval size and colouration, but also in the chaetotaxy of abdominal segment X and epicranium (presence or absence of seta Ea1), structural details of all the mouthparts, lengths of the several leg parts, and urogomphi. It is likely that the scale of the morphological differences between the larvae of species from one genus depends largely on their different ecological preferences. The above-mentioned *Haploglossa* species inhabit micro-environments (*H. picipennis* – nests of raptors, *H. nidicola* – nest holes of sand martins) that are very different from those of *Dinaraea* species, which are usually found under bark, mainly of broad-leaved trees.

Measurements of the head and pronotum of the two *Dinaraea* species indicate that their larval development involves three stages (Table 1): this is typical of most known aleocharines (White 1977, Ashe and Watrous 1984, Ashe 1985, Ashe 1986, Zagaja et al. 2014, Staniec et al. 2016). Only in the case of *Pella* species (*P. laticollis*) (Lomechusini), inhabiting ants’ nests, were just two larval stages found; this is due to the faster rate of development of these rove-beetles (Hölldobler et al. 1981, Staniec et al. 2009). This feature is probably an adaptation to the myrmecophilous lifestyle that aims to minimise the period during which staphylinid larvae are potentially endangered by their hosts in the anthill.

Morphological analysis of a *Dinaraea* larva revealed a series of differences between its first (L1), and its second (L2) and third (L3) instars, whose external structures are identical to that of L1. Apart from the clearly smaller body size (see Table 1), features exclusive to L1 include: (1) the absence of some setae on the dorsal surface of the head and thorax, and on the dorsal and ventral surfaces of the abdomen, (2) the presence of short subapical setae on the urogomphi, (3) egg bursters on some thoracic and abdominal tergites, (4) a darker terminal antennal segment than in L2–3, and (5) markedly longer urogomphi and their apical setae than in later stages. These morphological differences between the younger and older larval instars in *Dinaraea* are of a similar nature to those in other tribes of Aleocharinae (Table 2) (Ashe and Watrous 1984, Ashe 1986, Zagaja et al. 2014, Staniec et al. 2016). They enable one to easily distinguish L1 from the older larval stages without recourse to metric analysis. In view of the fragmentary nature of the available information on this subject, it is not possible to state definitively whether and to what extent these differences extend across the whole subfamily.

A combination of 17 diagnostic features (see “Generic diagnosis…”) have been proposed for the mature larva of *Dinaraea*, described above, which enable it to be distinguished from other known older larval stages of Aleocharinae (Paulian 1941, Pototskaya 1967, Topp 1975, Ashe 1981, Ashe and Watrous 1984, Ashe 1985, Ahn 1997, Jeon and Ahn 2009, Staniec et al. 2009, 2010, 2016, Zagaja et al. 2014). In this respect the genus Dalotia (Da) Casey, (Athetini) most closely resembles the genus Dinaraea (Di) (Ashe and Watrous 1984), and the small differences relate solely to: (i) body habitus – moderately dorso-ventrally flattened in *Di* or cylindrical in *Da*; (ii) shape of antennal article I longer than wide in *Di* or wider than long in *Da*; (iii) structure of anterior margin of labrum central region protruding and crenate in *Di* or wholly rounded and smooth in *Da*; (iv) seta Da1 on pronotum absent in *Di* or present in *Da*; (v) setae D3 and P5 on abdominal sternite I absent and present respectively in *Di*, or present and absent in *Da*. It should be added that the dorso-ventrally flattened body and moderately elongated antennal segment I are most probably adaptations to the under-bark lifestyle of this larva, as in the case of the mature form of *Dinaraea*. On the other hand, the structure of the anterior margin of the labrum, and particularly the specific features of the body chaetotaxy, may be of phylogenetic significance. That is why these structures have been included in the morphological descriptions of other Aleocharinae larvae (Ashe and Watrous 1984, Ashe 1985, 1986, Ahn 1997, Jeon and Ahn 2009, Staniec et al. 2009, 2010, 2017). Only a few of these species has the chaetotaxy not only of older but also of younger larval forms been described (Zagaja et al. 2014, Staniec 2016, the present study).

A preliminary comparative analysis of the features of the chaetotaxy was carried out on the basis of well-researched larvae of three different tribes of Aleocharinae. This revealed that all the larval stages of Athetini (*Dinaraea*) and Oxypodini (*Thiasophila*) are much more similar to one another than to the larvae of Homalotini (*Gyrophaena*) (Table 2). In the first two taxa the slight differences in chaetotaxy of L1–3 concern only the number and homology of the setae on the tergites and first sternite of the abdomen. By contrast, a distinctly smaller number of setae develop on all body tagmata of L1–3, especially the thoracic ones, in Homalotini than in the other two tribes. These data indicate the distinctly closer relationship of *Dinaraea* (Athetini) with *Thiasophila* (Oxypodini) than with *Gyrophaena* (Homalotini). This state is partially corroborated by Thomas (2009), whose research was based on the molecular analysis of two mitochondrial DNA markers. This author showed that Homalotini are a group separate from the other taxa he/she analysed. The taxa from Athetini and Oxypodini belong to sister groups, at least in some of the trees generated.

Another question relates to the significance of the features of chaetotaxy for phylogenesis depending on the larval stage. Ashe (1986) suggested that later developmental stages would be more useful at lower taxonomic levels (e.g. genus), as they exhibit more features associated with a better-developed chaetotaxy. In contrast, the first larval stages, with their smaller numbers of setae, could be phylogenetically significant in the analysis of higher systematic units. This hypothesis is convergent with that underlying our preliminary studies. These have shown that at the tribal level of Aleocharinae, distinct differences may occur in the chaetotaxy (e.g. between Athetini and Homalotini), between not only the late larval stages but also the early ones. Interestingly, in L1 of some species these differences may be even greater than in the older larval stages, e.g. the chaetotaxy of abdominal tergites I–VIII of *Dinaraea* and *Thiasophila* (Table 2).

It should also be borne in mind that the development of setae on the different body parts of the larvae during ontogenesis is uneven. In the taxa we are analysing here, the fewest setae appear on the head – two pairs at most, if any at all (*Gyrophaena*). In contrast, the changes in the chaetotaxy are the greatest on the pronotum, and somewhat less so on the abdominal tergites and sternites (Table 2). A homologous series of setae that appear in the older larval stages (L2–3) of species from the tribes Athetini [A], Oxypodini [O] and Homalotini [H]) has been established. On the thoracic tergites they are the following setae: A3, P3 (A, O, H) and Da3, Db3, Dc3, Dd1, L3 (A, O); on abdominal tergites I–VIII: P3 (O, A, H), Dc2 (O, A) and Db3 (O, H); on abdominal sternite I: D2 (A, O); on abdominal sternites II–VII: D2, P3 (A, O, H) and D3 (A, O); on abdominal sternite VIII: D2 (A, O, H) and D3, P3 (A, O). Being homologous aspects of the chaetotaxy, they could be of phylogenetic importance, especially at lower systematic levels, e.g. subtribe or genus, but this would have to be confirmed by further research.

As a representative of Athetini, the pupa of *Dinaraea* possesses general structural features, such as an exarate body type, lightly sclerotised, with numerous setae growing from basal, cuticular protuberances and double gonotheca on the ventral surface of the final segment in the female, characteristic of the pupal stages of other aleocharines from Homalotini, Lomechusinii, and Oxypodini (Ashe 1981, Staniec et al. 2009, 2010, Zagaja et al. 2014). Its specific appearance relating to the body outline, width of head, shape of pronotum, length of legs and antennae, shape, and length of mouthparts, resembles the features of the adult form.

As in *Dinaraea*, the production of a pupal, silken cocoon, into which particles of the surrounding substrate are often woven, has been observed in numerous tribes of Aleocharinae, such as Athetini, Aleocharini, Corotocini, Falagriini, Homalotini (including members of subtribes Bolitocharina, Gyrophaenina and Homalotina), Hypocyphtini, Liparocephalini, Lomechusini, Oxypodini, and Placusini (Ashe 1982, Frank and Thomas 1984, Thayer et al. 2004, Staniec et al. 2010, Zagaja et al. 2014). Some authors assume that this behaviour may be at least a basal condition of the higher classification of Aleocharinae. Topp (1975) even suggested that this feature occurs exclusively in this subfamily of Staphylinidae. However, this statement seems controversial in the light of reports from other researchers, who described a similar structure in taxa from the subfamilies Steninae and Staphylininae, although in the latter case, it is made mostly from soil (Weinreich 1968, Staniec 2004, Pietrykowska-Tudruj and Staniec 2007, Staniec et al. 2008).

The pupal cocoon undoubtedly plays a protective role. That is why it is probably so common in the Aleocharinae, including *Dinaraea*, in which the delicate exarate pupae are enclosed in a weakly sclerotised cuticle (Staniec et al. 2010, Zagaja et al. 2014, the present study). Likewise, in the case of some Staphylininae, whose larvae spin cocoons (e.g. *Gabrius splendidulus*, *Rabigus tenuis*), their pupae are covered by an exceptionally thin cuticle compared with other members of this subfamily (Pietrykowska-Tudruj and Staniec 2007, Staniec et al. 2008). Presumably, then, pupation within a cocoon could have evolved independently in members of many different subfamilies of a range of rove-beetles, as an adaptation associated with the pupal structure and/or the biotic conditions of the environment, e.g. pressure on the part of predators. Nevertheless, knowledge of the life history of Staphylinidae, including the occurrence of a pupal cocoon in this, the largest family of beetles, remains fragmentary and is restricted to a very small number of taxa summarised for Staphylinidae by Frank and Thomas (1984). Thus, more data are required before more binding conclusions regarding this structure can be drawn.

In Europe, both *Dinaraea* species are quite widespread saproxylic rove-beetles, although both in Poland and some other countries *D. linearis* appears to be far less common than *D. aequata*. The flattened and parallel-sided body of adults and larvae (the present study) of these staphylinids are probably a consequence of their mode of life under tree bark; they do not display any particular preferences as regards the species of tree they colonise. *D. aequata* is also encountered in various arboreal fungi. In Poland both these rove-beetle species can be observed in nature all the year round, along with some 30 other species of saproxylic Coleoptera, from six families (Nikitsky and Schigel 2004, Alexander and Anderson 2012, Melke, oral information; authors’ unpublished data).

In the rearing, the development of *D. aequata* and *D. linearis* took place in the autumn and winter months (November–February), as in the case of *Phloeonomus punctipennis* Thomson (Omaliinae) – another saproxylic staphylinid, whose larval stages were caught in the field in the second half of November (Staniec et al. 2016). It is not exactly known, however, whether the above-mentioned reproductive period of *Dinaraea* in the laboratory coincided in time with its reproduction in nature, or whether it was more the effect of the suitable ambient temperature at which the rearings were performed. Certain information was also obtained as regards the diet of these rove beetles, which was hitherto completely unknown (Klimaszewski et al. 2013). On the basis of laboratory observations, these rove beetles are presumably predators, which in natural conditions hunt for various tiny arthropods with delicate cuticles and consume their soft tissues.

## References

[B1] Ahn K (1997) A review of Liparocephalus Mäklin (Coleoptera: Staphylinidae: Aleocharinae) with descriptions of larvae. Pan-Pacific Entomologist 73: 79–92.

[B2] Alexander KNA Anderson R (2012) The beetles of decaying wood in Ireland – A provisional annotated checklist of saproxylic Coleoptera. Irish Wildlife Manuals, No. 65, National Parks and Wildlife Service, Department of the Arts, Heritage and the Gaeltacht, Dublin, 165 pp.

[B3] Ashe JS (1981) Studies of the life history and habits of *Phanerota fasciata* Say (Coleoptera: Staphylinidae: Aleocharinae) with notes on the mushroom as a habitat and descriptions of the immature stages. Coleopterists’ Bulletin 35: 183–96.

[B4] Ashe JS (1982) Construction of pupal cells by larvae of Aleocharinae (Coleoptera: Staphylinidae). Coleopterists’ Bulletin 35: 341–343.

[B5] Ashe JS (1985) Fecundity, development and natural history of *Meronera venustula* (Erichson) (Coleoptera: Staphylinidae: Aleocharinae). Psyche 92: 181–204. https://doi.org/10.1155/1985/10417

[B6] Ashe JS (1986) Structural features and phylogenetic relationships among larvae of genera of gyrophaenine staphylinids (Coleoptera: Staphylinidae: Aleocharinae). Fieldiana, Zoology (NS) 30: 1–60.

[B7] Ashe JS (2005) Phylogeny of the tachyporine group of subfamilies and ‘basal’ lineages of the Aleocharinae (Coleoptera: Staphylinidae) based on larval and adult characteristics. Systematic Entomology 30: 3–37. https://doi.org/10.1111/j.1365-3113.2004.00258.x

[B8] Ashe JS Watrous LE (1984) Larval chaetotaxy of Aleocharinae (Staphylinidae) based on a description of *Atheta coriaria* Kraatz. Coleopterists’ Bulletin 38: 165–179.

[B9] Benick G Lohse GA (1974) 22. U.F.: Aleocharinae. In: Freude H Harde KW Lohse GA (Eds) Die Käfer Mitteleuropas. Band 5. Staphylinidae II (Hypocyphtinae und Aleocharinae), Pselaphidae. Goecke & Evers, Krefeld, 115–116.

[B10] Burakowski B Mroczkowski M Stefańska J (1981) Chrząszcze Coleoptera-Staphylinidae, p. 3. Katalog Fauny Polski, p. XXIII. Vol. 8. Polskie Wydawnictwo Naukowe, Warszawa, 330 pp.

[B11] Frank JH Thomas MC (1984) Cocoon-spinning and the defensive function of the median gland in larvae of Aleocharinae (Coleoptera: Staphylinidae): a review. Quaestiones Entomologicae 20: 7–23.

[B12] Grebennikov VV Newton AF (2009) Good-bye Scydmaenidae: or why the ant-like stone beetles should become megadiverse Staphylinidae sensu latissimo (Coleoptera). European Journal of Entomology 106: 275–301. https://doi.org/10.14411/eje.2009.035

[B13] Hölldobler B Möglich M Maschwitz U (1981) Myrmecophilic Relationship of *Pella* (Coleoptera: Staphylinidae) to *Lasius fuliginosus* (Hymenoptera: Formicidae). Psyche 88: 347–374. https://doi.org/10.1155/1981/75317

[B14] Jeon MJ Ahn KJ (2009) Description of late-instars of *Bryothinusa koreana* Ahn and Jeon (Coleoptera: Staphylinidae: Aleocharinae) by association of life stage based on DNA sequence data. Florida Entomologist 92: 367–373. https://doi.org/10.1653/024.092.0224

[B15] Klimaszewski J Webster RP Langor DW Bourdon C Jacobs J (2013) Review of Canadian species of the genus *Dinaraea* Thomson, with descriptions of six new species (Coleoptera, Staphylinidae, Aleocharinae, Athetini). ZooKeys 327: 65–101. https://doi.org/10.3897/zookeys.327.5908 10.3897/zookeys.327.5908 PMC3804766 24167422

[B16] Koch K (1989) Die Käfer Mitteleuropas. Ökologie, 1. Goecke & Evers Verlag, Krefeld, 440 pp.

[B17] Löbl I Löbl D (2015) Catalogue of Palaearctic Coleoptera. Hydrophiloidea-Staphylinoidea. Vol. 2. Revised and updated edition. Brill, Leiden, Boston, 702 pp.

[B18] Newton AF Thayer MK Ashe JS Chandler DS (2000) Family 22. Staphylinidae Latreille, 1802. In: Arnett RHJr Thomas MC (Eds) American Beetles, Volume 1, Archostemata, Myxophaga, Adephaga, Polyphaga: Staphyliniformia. CRC Press LLC, Boca Raton, FL, 272–418.

[B19] Nikitsky NB Schigel DS (2004) Beetles in polypores of the Moscow region: checklist and ecological notes. Entomologica Fennica 15: 6–22.

[B20] Paulian R (1941) Les premiers états des Staphylinoidea. Mémoires du Muséum national d’histoire naturelle 15: 1–361.

[B21] Pietrykowska-Tudruj E Staniec B (2007) The pupae of *Gabrius splendidulus* (Gravenhorst, 1802) and *Neobisnius procerulus* (Gravenhorst, 1806) (*Coleoptera*: *Staphylinidae*). International Journal of Invertebrate Taxonomy – Genus 18: 351–358.

[B22] Pietrykowska-Tudruj E Staniec B Solodovnikov A (2012) Discovery of the *Quedius antipodum* Sharp larva from New Zealand: phylogenetic test of larval morphology for Staphylinini at the intratribal level (Coleoptera: Staphylinidae). Systematic Entomology 37: 360–378.

[B23] Pietrykowska-Tudruj E Staniec B Wojas T Solodovnikov A (2014) Immature stages and phylogenetic importance of *Astrapaeus*, a rove beetle genus of puzzling systematic position (Coleoptera, Staphylinidae, Staphylinini). Contributions to Zoology 83(1): 41–65. https://doi.org/10.1111/j.1365-3113.2011.00612.x

[B24] Pototskaya VA (1967) Opredelitel’ lichinok korotkonadkrylykh zhukov evropeiskoi chasti SSSR. Academiya Nauk SSSR, Izdatel’stvo Nauka, Moskva, 120 pp.

[B25] Staniec B (2004) The pupae of *Ontholestes murinus* (Linnaeus, 1758), *Philonthus rectangulus* Sharp, 1874 and a supplement to the pupal morphology of *Philonthus succicola* Thomson, 1860 (*Coleoptera*: *Staphylinidae*). International Journal of Invertebrate Taxonomy – Genus 15: 37–46.

[B26] Staniec B Pietrykowska-Tudruj E (2008) Immature stages of *Rabigus tenuis* (Fabricius, 1792) (Coleoptera, Staphylinidae, Staphylininae) with observation on its biology and taxonomic comments. Belgian Journal of Zoology 138(1): 22–39.

[B27] Staniec B Pietrykowska-Tudruj E Pilipczuk J (2009) Morphology of the developmental stages of *Pella* (= *Zyras*) *laticollis* (Märkell, 1844) with remarks on its biology (Coleoptera: Staphylinidae). Genus 20: 225–242.

[B28] Staniec B Pietrykowska-Tudruj E Zagaja M (2010) Description of the larva and pupa of *Haploglossa picipennis* (Gyllenhal, 1827) and larva of *H. nidicola* (Fairmaire, 1852) (Coleoptera, Staphylinidae, Aleocharinae) with taxonomic remarks. Entomologica Fennica 21: 151–167.

[B29] Staniec B Pietrykowska-Tudruj E Czepiel-Mil K (2016) Larva of *Gyrophaena boleti* (Linnaeus, 1758) (Coleoptera: Staphylinidae) – an obligatory saproxylic and mycophagous species associated with *Fomitopsis pinicola*: notes on tergal gland system and behavior. Annales Zoologici 66(1): 83–100. ttps://doi.org/10.3161/00034541ANZ2016.66.1.006

[B30] Staniec B Pietrykowska-Tudruj E Wagner K (2016) Ultramorphological characteristics of unknown larva of *Phloeonomus punctipennis* Thomson, 1867 (Coleoptera; Staphylinidae; Omaliinae) – an obligate saproxylic species: notes on chaetotaxy and ecological preferences. Zootaxa 4171(3): 475–490. https://doi.org/10.11646/zootaxa.4171.3.4 27701212 10.11646/zootaxa.4171.3.4

[B31] Topp W (1975) Zur Larvalmorphologie der Athetea (Col., Staphylinidae). Stuttgarter Beiträge Zur Naturkunde Ser. A (Biologie) 268: 1–23.

[B32] Topp W (1978) Bestimmungstabelle für die Larven der Staphylinidae, In: Klausnitzer B (Ed.) Ordnung Coleoptera (Larven). Dr. W. Junk Publishers, The Hague, 304–334.

[B33] Weinreich E (1968) Über den Klebfangapparat der Imagines von *Stenus* Latr. (Coleopt.; Staphylinidae) mit einem Beitrag zur Kenntnis der Jugendstadien dieser Gattung. Zeitschrift für Morphologie und Ökologie der Tiere 62: 162–210. https://doi.org/10.1007/BF00299123

[B34] White IM (1977) The larvae of some British species of *Gyrophaena* Mannerheim (Coleoptera: Staphylinidae) with notes on the taxonomy and biology of the genus. Zoological Journal of the Linnean Society of London 60(4): 283–318. https://doi.org/10.1603/0013-8746(2004)097[0624:DOTRLO]2.0.CO;2

[B35] Thayer MK Ashe JS Hanley RS (2004) Discovery of the remarkable larvae of Hoplandriini (Coleoptera: Staphylinidae: Aleocharinae). Annals of the Entomological Society of America 97: 624–634. http://dx.doi.org/10.1603/0013–8746(2004)097[0624:dotrlo]2.0.co;2

[B36] Thomas JC (2009) A preliminary molecular investigation of aleocharine phylogeny (Coleoptera: Staphylinidae). Annals of the Entomological Society of America 102: 189–195. https://doi.org/10.1603/008.102.0201

[B37] Zagaja M Staniec B Pietrykowska-Tudruj E (2014) The first morphological description of the immature stages of *Thiasophila* Kraatz, 1856 (Coleoptera; Staphylinidae) inhabiting ant colonies of the *Formica rufa* group. Zootaxa 3774(4): 301–323. https://doi.org/10.11646/zootaxa.3774.4.1 24871503 10.11646/zootaxa.3774.4.1

